# Hospital Meetings

**Published:** 1904-02-06

**Authors:** 


### HOSPITAL MEETINGS.

### THE ITALIAN HOSPITAL.

THE Annual General Meeting of Governors of this hospital was held on Saturday, January 30th, the Marquis Carignani di Novoli, Chargé d'Affaires, presiding in the absence of the Italian Ambassador.

A resolution tendering the thanks of the Governors and Committee of Management to the King of Italy for his recent gift of £1,000 was unanimously adopted by the meeting, as was also a similar vote to his Royal Highness the President of King Edward's Hospital Fund, for the yearly grant of £500 awarded from the fund to keep open ten beds. This resolution, which was proposed by the Chairman, was seconded by Lord Edmund Talbot, M.P., who said they were all aware what a great interest his Royal Highness took in hospital work in this country, and their thanks were especially due to him for the keen and genuine personal interest he took in their hospital.

The adoption of the report was then moved by the Marquis Carignani di Novoli and seconded by Cav. Luciano de Rin Hon. Secretary, who pointed out that although the in-patients of the hospital had increased by 11 per cent., the out-patients by 32 per cent., yet their expenditure had only increased by £515 per annum; unfortunately their receipts had fallen off by £1,000, and he hoped th Governors would endeavour to induce their friends to make further donations.

A vote of thanks to the Chairman closed the proceedings.

The report for 1903 (only part of which has as yet been published) records a large increase in work in both in and out-patients' departments, the total number in each being the largest in any one year since the foundation of the hospital. The annual grant from King Edward's Fund had enabled them to throw open all their beds. The total of patients treated is 14,013, as against 10,711 in 1902, and the ordinary expenditure is £2,866 against £2,351, while the ordinary income is £2,532 against £3,419. The committee had decided to utilise the King of Italy's gift of £1,000, which was sent "as a special mark of his Majesty's approval of the hospital's work, and of his regret that time did not allow his Majesty to visit the institution personally," in endowing a bed to be called the "Queen Elena" bed, and the money had been invested for that purpose. During the year 669 in-patients and 13,344 out-patients received treatment, of which 6,685 were British subjects, 5,563 Italians, and 1,765 representatives of other nationalities.

The committee make an earnest appeal for increased assistance, and draw attention to the forthcoming annual festival ball, to be held at the Royal Institute of Painters in Water Colours, on May 9th.

### GENERAL LYING-IN HOSPITAL.

THE annual meeting of Governors of this hospital was held on Wednesday, the 27th ult, Lord Saye and Sele presiding.

The Rev. A. W. Jephson, in moving the adoption of the report and balance-sheet for 1903, remarked that the difficulties of up keep at this institution, as at other modern London hospitals, tended rather to increase than diminish. The excess of expenditure over income, amounting to £363, was due to an increase in the payment of the out-door midwives' fees, the higher cost of provisions, and the requirements of modern surgical science; while a debt had been unavoidably incurred for sanitary improvements, repairs, and an entirely new drainage system. The deficit had occurred notwithstanding the exertions of the present Secretary (Miss Rose E. Whyte), under whose care the subscriptions had increased; and although its existence might arouse the sympathies of those dispensers of funds who considered that the more a hospital was in debt, the greater was its claim for assistance, yet it was a source of anxiety to the committee, more especially as they had always prided themselves upon making both ends meet. A glance at the subscription list showed how little local support the hospital received, and this notwithstanding the fact that it had been carrying on an eminently useful work in the midst of a crowded neighbourhood for 150 years. The tokens of interest given during the year by the Patrons, H.M. Queen Alexandra and H.R.H. the Princess of Wales, were a source of great encouragement. The committee had gladly welcomed the Visitors of King Edward's Hospital Fund, and were pleased to be able to announce a donation from the Fund of £200. It was with deep regret that the committee learnt that the generous donor of the Convalescent Home at Woking found herself unable to continue to maintain it at her sole expense. The home had proved to be so great a boon to the patients, that the committee trusted the efforts now being made to carry it on by means of private subscriptions would be attended with success.

The medical report was presented by Dr. W. R. Dakin, who said that, owing to the large number of applications for admission to the hospital during the year, the available accommodation had been at times severely taxed, while the number of women attended in their own homes showed an increase of 346 on that of the previous year. Applications for training were also far in excess of the number that could be received, while the demand for both nurses and midwives trained in the institution was very encouraging. Eighty-one nurses and 24 midwives had passed through the school during 1903; and of the latter, 18 had obtained the certificates of the London Obstetrical Society, while the remaining six were awaiting examination. Of the 556 patients received, three had died, while 530 children left the hospital alive and well.

The reports having been adopted, Mr. Malkin proposed a vote of thanks to the Management Committee, Medical Staff, and Auditors. It was the first time he had had to condole with the committee on the bad state of the finances; and as representing the Governors, he wished to say how deeply they sympathised, and how sincerely they hoped the burden of debt would soon be lightened. If there were any justice in the world, these sordid anxieties would, he thought, soon be removed.

Dr. Cullingworth, having seconded the motion, proceeded to pay a high tribute to the quality of the year's work. The hospital, he said, was an example to the kingdom of how the process of parturition could be made practically free from appreciable risk. From that point alone, the value of the work done in this hospital, since its reorganisation on antiseptic lines, was quite incalculable; there was no break in the record of freedom from septic poisoning. The report presented by Dr. Dakin was as good as could be desired; for while it was impossible to eliminate all sources of mortality in work of this kind, the efforts of the staff had been attended with a high degree of success, and the thanks of the Governors were thoroughly well deserved.

Dr. F. H. Champneys proposed that Lord Lister be asked to continue to hold the office of president; his absence from this annual meeting, owing to ill-health, was a source of great regret to the committee. Mr. Merivale seconded, and the motion was carried.

The re-election of other officers was then proceeded with, after which Mr. Maudslay proposed, and Mr. Owen seconded, a vote of thanks to the chairman. In reply, Lord Saye and Sele said he was rejoiced to have the opportunity of rendering a service to the institution. A lying-in hospital, he thought, appealed to the emotions as no other did; he felt it a great privilege to be associated in a small degree with men who were devoting time and skill to the objects for which that unpretending little hospital was founded.

